# Draft Genome Sequences of Three Pasteurella multocida Strains Isolated from Domestic Animals in Kazakhstan

**DOI:** 10.1128/MRA.00487-20

**Published:** 2020-08-06

**Authors:** Asylulan Amirgazin, Gilles Vergnaud, Kasym Mukanov, Marat Kuibagarov, Talgat Karibaev, Yerlan Ramankulov, Alexandr Shevtsov

**Affiliations:** a National Center for Biotechnology, Nur-Sultan, Kazakhstan; b Institute for Integrative Biology of the Cell (I2BC), Université Paris-Saclay, Gif-sur-Yvette, France; c National Reference Veterinary Center, Nur-Sultan, Kazakhstan; d School of Science and Technology, Nazarbayev University, Nur-Sultan, Kazakhstan; University of Southern California

## Abstract

We report here the draft genome sequences of three strains of Pasteurella multocida isolated in Kazakhstan from domestic animals that died due to hemorrhagic septicemia.

## ANNOUNCEMENT

Pasteurella multocida is a Gram-negative bacterium causing pasteurellosis, which is most dangerous in the form of hemorrhagic septicemia (HS). In Kazakhstan, pasteurellosis is an endemic infection, with sporadic cases among domestic animals and pandemic outbreaks among saiga populations. The case fatality rate due to HS can reach 90% of the animal population. An example of this is the outbreaks between 2008 and 2015, which led to a catastrophic decline in saiga populations, putting them on the brink of extinction (1). Information on the genetic diversity of P. multocida circulating in Kazakhstan among wild and particularly domestic animals is limited (2, 3). The goal of this work was to improve our knowledge of P. multocida strains circulating in domestic animals in Kazakhstan.

Three strains of P. multocida were deposited in the collection of the National Reference Veterinary Center. The strains P-mult-5-KZ, P-mult-15-KZ, and P-mult-10-KZ were isolated in 2006, 2015, and 2013, in the Kostanay, Almaty, and East Kazakhstan regions of Kazakhstan, from pathological material from two horses and cattle, respectively. Isolates were recovered by seeding a 10% suspension of the liver and spleen onto nutrient agar (HiMedia, India) for 24 h at 37°C. Single colonies were subcultured in a nutrient broth (HiMedia) for 24 h at 37°C. The culture of the P-mult-5-KZ strain was stored in a lyophilized state, followed by a repeat of the procedures described above. DNA was isolated from the accumulated cultures using the QIAamp DNA minikit (Qiagen, USA), with subsequent 16S rRNA gene identification (4), and then the DNA was stored at −70°C. These strains are no longer available from the National Reference Veterinary Center.

DNA libraries were prepared using the Nextera XT DNA library prep kit (Illumina, San Diego, CA, USA), according to the manufacturer’s instructions. Sequencing was performed using a reagent kit v3 (2 × 300 bp) on the MiSeq system (Illumina). The raw reads were quality controlled using FastQC v0.11.9 (5) and trimmed using Geneious Prime v2019.2 with BBDuk trimmer plugin v1.0. The reads were assembled using SKESA v2.3.0 (6). The assembly quality was evaluated using QUAST v5.0.2 (7). Identification of capsular and lipopolysaccharide loci was carried out by performing a contig search with BLAST+ v2.9.0 and confirmed by multiplex PCR (8, 9). Identification of sequence types (STs) was carried out using the Pasteurella multocida multihost multilocus sequence type (MLST) database (http://pubmlst.org/pmultocida/multihost/introduction.shtml) and the BioNumerics MLST plugin (Applied Maths, Sint-Martens-Latem, Belgium). Genome annotation was performed using the NCBI Prokaryotic Genome Annotation Pipeline (PGAP) v4.11 (10, 11). Default parameters were used for all software.

The assembly characteristics and genotypes are presented in Table 1. Genotyping of the capsular, lipopolysaccharide, and multihost MLST loci showed that all three strains belong to the B:L2:multihost ST64 genotype. The genotyping results are similar to the previously described data in the study by Orynbayev et al. (2). Therefore, it can be judged that the B:L2:multihost ST64 is a major characteristic genotype of the P. multocida strains circulating in Kazakhstan. The obtained sequence data will be helpful for revealing the genetic differences responsible for the virulence and pathogenicity of the hemorrhagic septicemia-associated strains of P. multocida.

**TABLE 1:** Characteristics and accession numbers of P. multocida genomes

Strain	Genotype	Total no. of reads	Genome size (bp)	No. of contigs	Coverage (×)	*N*_50_ (bp)	G+C content (%)	No. of genes	GenBank accession no.	SRA accession no.
P-mult-5-KZ	B:L2:ST64	318,693	2,256,412	29	32.69	237,868	40.28	2,146	JAAONW000000000	SRR11293610
P-mult-10-KZ	B:L2:ST64	365,028	2,300,365	30	37.18	382,962	40.25	2,200	JAAILE000000000	SRR9841486
P-mult-15-KZ	B:L2:ST64	225,053	2,255,675	28	25.81	220,435	40.29	2,146	JAAONX000000000	SRR11293647

### Data availability.

This whole-genome shotgun project has been deposited in DDBJ/ENA/GenBank under accession numbers JAAONW000000000, JAAILE000000000, and JAAONX000000000. The raw data from BioProject PRJNA556768 were submitted to the NCBI SRA under accession numbers SRR11293610, SRR9841486, and SRR11293647.
